# Acute Polyserositis with Cardiac Tamponade and Bilateral Refractory Pleural Effusion after ChAdOx1 nCoV-19 Vaccination

**DOI:** 10.3390/vaccines10081286

**Published:** 2022-08-10

**Authors:** Guan-Yi Li, Chang-Ching Lee, Chin-Chou Huang

**Affiliations:** 1 Division of Cardiology, Department of Medicine, Taipei Veterans General Hospital, Taipei 112201, Taiwan; 2 Department of Chest Medicine, Taipei Veterans General Hospital, Taipei 112201, Taiwan; 3 School of Medicine, National Yang Ming Chiao Tung University, Taipei 112304, Taiwan; 4 Cardiovascular Research Center, National Yang Ming Chiao Tung University, Taipei 112304, Taiwan

**Keywords:** cardiac tamponade, ChAdOx1 nCoV-19, COVID-19, pleural effusion, polyserositis, vaccine

## Abstract

The association of SARS-CoV-2 messenger ribonucleic acid vaccines with pericarditis in young adults has been reported. However, data regarding other types of vaccines are extremely limited. We presented a 94-year-old man with rapidly progressive dyspnea and fatigue six days after his first ChAdOx1 nCoV-19 vaccination. Impending cardiac tamponade and bilateral pleural effusion were found. Hence, massive yellowish pericardial and pleural effusion were drained. However, the pleural effusion persisted and pigtail catheters were inserted bilaterally. After serial studies including surgical pleural biopsy, acute polyserositis (pericarditis and pleurisy) was diagnosed. Anti-inflammatory treatment with colchicine and prednisolone was administered. All effusions resolved accordingly. This rare case sheds light on the presentation of ChAdOx1 nCoV-19 vaccine-related acute polyserositis. In conclusion, awareness of this potential adverse event may facilitate the diagnosis for unexplained pericardial or pleural effusion after vaccination.

## 1. Introduction

For the current worldwide SARS-CoV-2 pandemic, vaccination plays a crucial role in preventing its life-threatening complications. Despite the fact that the benefit of the SARS-CoV-2 vaccine outweighs the risk, more and more adverse events have been reported. The association of SARS-CoV-2 messenger ribonucleic acid (mRNA) vaccines (Pfizer-BioNTech and Moderna) with pericarditis and myocarditis in young adults has been reported, particularly for male adolescents [1,2,3,4]. However, data regarding other types of coronavirus disease 2019 (COVID-19) vaccines, such as the viral vector vaccines, are extremely limited.

We presented an old man who developed acute polyserositis complicating with cardiac tamponade and refractory bilateral pleural effusion (PE) within six days after receiving ChAdOx1 nCoV-19 (AstraZeneca) vaccination. We believe this case could shed light on the presentation of the rare ChAdOx1 nCoV-19 vaccine-related acute polyserositis.

## 2. Case Presentation

A 94-year-old man presented with acute onset, rapidly progressive dyspnea and fatigue after his first ChAdOx1 nCoV-19 vaccination. His symptoms began on the second day after vaccination. Initially, he did not take medicine or seek medical treatment. However, his condition deteriorated continuously, so he was brought to our emergent department on the sixth day.

In the past, he lived independently. He had the medical histories of hypertension and hyperlipidemia, and he took amlodipine and olmesartan regularly. Reviewing his medical record, his office systolic blood pressure was around 140–160 mmHg, while his office diastolic blood pressure was around 55–70 mmHg. Otherwise, he had no comorbidities such as heart failure, coronary artery disease, renal disease, cirrhosis, autoimmune disease, or malignancy.

**Figure 1:**
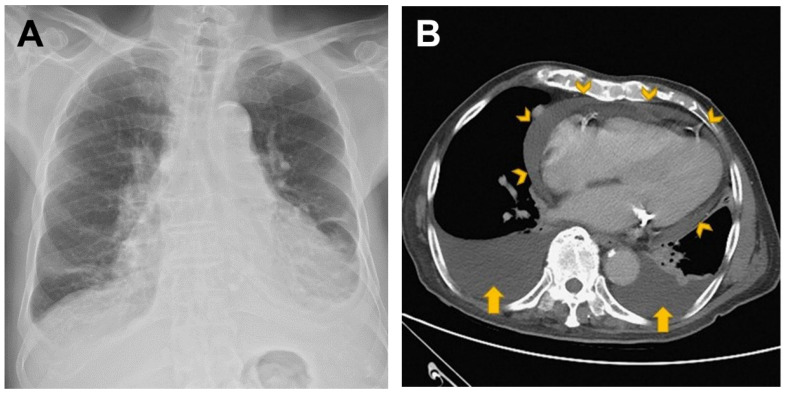

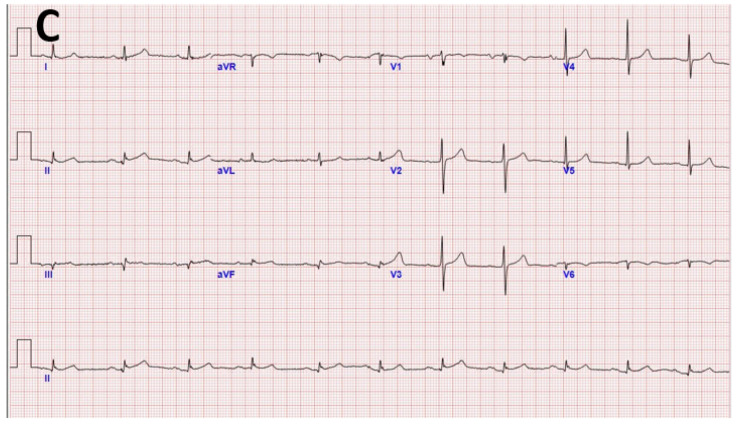
Acute polyserositis with cardiac tamponade and bilateral refractory pleural effusion after ChAdOx1 nCoV-19 vaccination. (**A**) Chest X-ray shows the typical water bottle sign, which refers to the shape of the cardiac silhouette in patients who have a large pericardial effusion. The fluid causes the pericardium to sag, mimicking an old-fashioned water bottle sitting on the bench. (**B**) Computed tomography reveals the presence of massive pericardial effusion (arrowheads) and bilateral pleural effusion (arrows). (**C**) A 12-lead electrocardiogram shows sinus rhythm with low QRS voltage, suggesting massive pericardial effusion.

**Figure 2:**
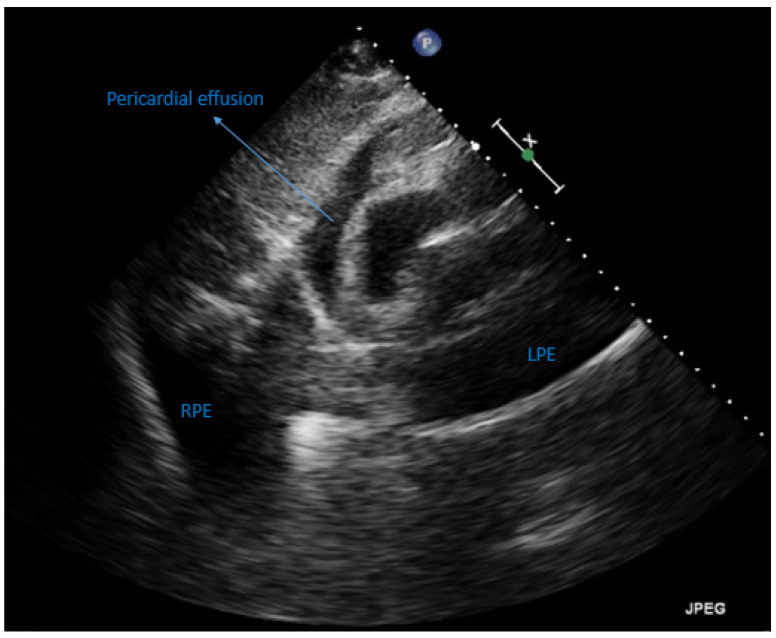
Transthoracic echocardiography captured on the 45th day during hospitalization. The subcostal view shows a small amount of pericardial effusion and massive refractory pleural effusion bilaterally. LPE = left pleural effusion; RPE = right pleural effusion.

Upon arriving at the emergency department, the patient was hypotensive (99/51 mmHg) and oxygen desaturated (94%, under nasal cannula with 3-L of oxygen per minute). He was afebrile (35.5 degrees Celsius) and had a pulse rate of 61 beats per minute, and a respiratory rate of 22 breaths per minute. A physical examination revealed a distant heart sound and decreased breathing sounds over bilateral lower lung fields. Laboratory data were unremarkable, including white blood cell count (4500/uL, normal range: 4180–9380/uL); blood urea nitrogen (26 mg/dL, normal range: 7–30 mg/dL); creatinine (1.11 mg/dL, normal range: 0.7–1.2 mg/L); C-reactive protein (0.19 mg/dL, normal range < 0.5 mg/dL); procalcitonin (0.05 ng/mL, normal range < 0.05 ng/mL); and N-terminal pro-brain natriuretic peptide (172 pg/mL, normal range < 450 pg/mL). A nasopharyngeal swab reverse transcription polymerase chain reaction test for COVID-19 was negative. Chest X-ray revealed the water bottle sign and bilateral costophrenic angle blunting (Figure 1A). Subsequent computed tomography confirmed the presence of pericardial effusion and bilateral PE (Figure 1B). The 12-lead electrocardiogram showed sinus rhythm but low QRS voltage (Figure 1C). Echocardiography revealed preserved left and right ventricular systolic function but impending cardiac tamponade. Hence, 400 mL of yellowish pericardial effusion was drained, as well as 620 mL of left PE. Albumin and diuretics were administered to maintain a negative fluid balance. Empiric antibiotics were also prescribed because parapneumonic PE could not be completely ruled out. However, echocardiography on the 45th day still revealed a small amount of pericardial effusion and massive refractory PE bilaterally (Figure 2). Therefore, pigtail catheters were inserted bilaterally.

**Figure 3:**
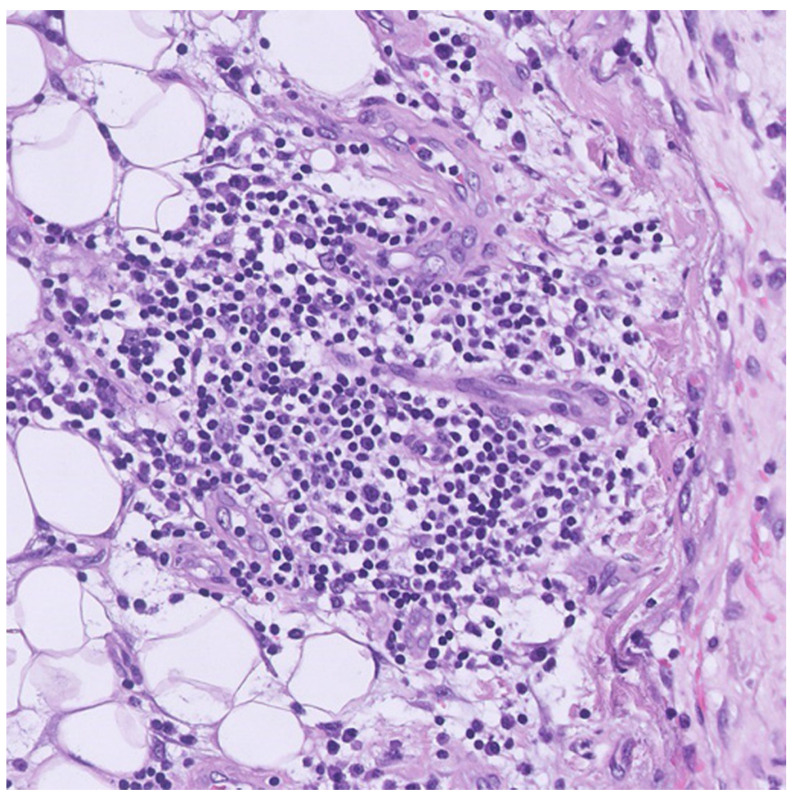
Microscopic view of the pleural specimen. Hematoxylin and eosin-stained pleural tissue by surgical biopsy shows focal lymphocytic infiltration, but without the presence of malignancy, granuloma, or lupus erythematosus cells. Original magnification 20×.

All pericardial effusion and PE were lymphocytes-predominant exudates and polymorphonuclear leukocytes could be yielded from them, serving as evidence of local inflammation of the pericardium and the pleura. Serositis, namely the inflammation of the serous tissue, might be attributed to numerous etiologies including infection, malignancy, uremia, or autoimmune disease, while some of them are idiopathic. The serial effusion culture for bacterium, fungus, and tuberculosis showed negative findings, except for one episode of secondary infection with *Staphylococcus aureus* yielded from the left pigtail during hospitalization, which subsided soon after antibiotic therapy. The polymerase chain reaction test of the blood and nasopharyngeal swab for various cardiotropic viruses were all negative. Combined with the initially normal white blood cell count, low level of C-reactive protein and procalcitonin, it made a primary infectious etiology less likely. Moreover, the tumor markers were within a normal range, and the cytology of all effusions did not yield any malignant cell. None of the image studies demonstrated a primary tumor or the pleural/pericardial seeding. Surgical pleural biopsy revealed inflammation with focal lymphocytic infiltration, but without the presence of malignancy, granuloma, or lupus erythematosus cells (Figure 3). His renal function was preserved, and the blood urea nitrogen level was relatively low during the whole course of hospitalization, which excluded the uremic serositis. His thyroid function was normal, and examinations for autoimmune disease were inconclusive. The remarkable temporal correlation between vaccination and the onset of symptoms made the inflammation unlikely to be attributed to an underlying autoimmune disease.

**Figure 4:**
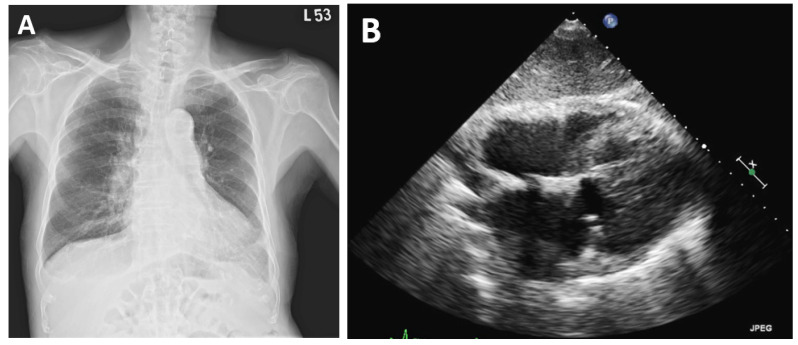
Repeated image studies during follow-up at the outpatient clinic. Chest X-ray (**A**) and the subcostal view of transthoracic echocardiography (**B**). Both show no recurrence of the effusion.

Vaccine-induced polyserositis (pericarditis and pleurisy) was diagnosed by exclusion. Anti-inflammatory treatment with colchicine and prednisolone was administered. All effusions resolved accordingly, and the pigtail catheters were removed. The patient was discharged home without complications. Until now, the patient had a regular follow-up at the outpatient clinic for about 1 year. Repeated chest X-ray and sonography showed no recurrence of the effusion (Figure 4A,B).

## 3. Discussion

Polyserositis is the inflammation with effusion of different serous membranes at the same time. A variety of vaccines including those for smallpox, influenza, and pneumococcus have been reported to be associated with pericarditis or polyserositis [5,6,7]. For the SARS-CoV-2 pandemic, different types of vaccine are currently available. Increasing concerns about pericarditis and/or myocarditis following the mRNA vaccine injection have been raised [1,2,3,4]. Of note, COVID-19 vaccine-related cardiac involvement usually presents in young men [8,9,10]. Moreover, data regarding the association between ChAdOx1 nCoV-19 vaccines, classified as a viral vector vaccine, with either perimyocarditis or pleurisy are extremely limited.

A variety of COVID-19 vaccines including ChAdOx1, mRNA1273, BNT162b2, MVC-COV1901, and NVX-CoV2373 are currently available in Taiwan. According to the latest data from the Taiwan Centers for Disease Control, the vaccination coverage rate in Taiwan is high (first, second, and booster doses: 91.8%, 85.8%, and 71.2%, respectively). More than 15 million ChAdOx1 nCoV-19 vaccines have been injected. As per the national Vaccine Adverse Event Reporting System, a total of 384 cases were reported for suspected pericarditis or myocarditis. The median onset of these events is 3 days following vaccination. Twenty-three out of the 384 cases (6.0%) were reported after ChAdOx1 nCoV-19 vaccination. To the best of our knowledge, this is the first case report of acute polyserositis complicating with cardiac tamponade and bilateral refractory PE after receiving the ChAdOx1 nCoV-19 vaccination in an old patient without an underlying autoimmune disease.

We made the diagnosis of vaccine-induced polyserositis by exclusion. First, neither malignant cells nor pathogens, including bacterium, fungus, virus, or tuberculosis could be identified, except for one episode of secondary infection with *Staphylococcus aureus*. The patient had no family history or past history of an autoimmune disease. The onset of autoimmune disease at this very old age should be rare. The patient had no typical presentation of autoimmune diseases such as skin rash, muscle weakness, skin thickening, telangiectasia, oral ulcer, arthralgia, or Raynaud’s phenomenon. As the tests for autoantibodies were inconclusive, he could not be classified with a specific autoimmune disease by the current criteria.

In addition to polyserositis, hypothyroidism and congestive heart failure are other differential diagnoses for a concomitant pericardial and pleural effusion [11]. Nevertheless, the patient’s thyroid function was normal, and the effusions associated with the increased filling pressure are usually transudate. The patient had a low level of N-terminal pro-brain natriuretic peptide. Moreover, serial echocardiography during the hospitalization all showed preserved left and right ventricle systolic function, making the heart failure related effusion improbable.

Although the pathological examination by surgical pleural biopsy cannot be the definite evidence for vaccine-induced polyserositis, it is helpful to exclude other etiologies. It revealed focal lymphocytic infiltration without the presence of malignancy, granuloma, or lupus erythematosus cells. It made the differential diagnosis of malignant effusion, tuberculosis, and systemic lupus erythematosus less likely. Combined with all the above-mentioned evidence, as well as the strong temporal association between vaccination and the symptoms, a diagnosis of vaccine-induced polyserositis could be made.

Since the relation between his illness and COVID-19 vaccination is only made by exclusion, future serologic experiments such as antibody response are still indicated. They may not only assess the safety and reactogenicity of the ChAdOx1 nCoV-19 vaccine, but also serve as the evidence of post-vaccination adverse reaction [12]. It is worth noting that pericardial or pleural effusion may be one sign of SARS-CoV-2 infection [13,14,15]. According to a systematic review, the cardiac complications and pathological features following mRNA COVID-19 vaccination were very similar to SARS-CoV-2 infection [16]. Further immunophenotyping studies investigating potential mechanisms of vaccine-mediated polyserositis are necessary to determine if it shares a similar pathophysiology with the effusions caused by the COVID-19 virus itself, as well as the population at higher risk for this adverse event.

## 4. Conclusions

This case sheds light on the presentation of ChAdOx1 nCoV-19 vaccine-related acute polyserositis. Recognition of this potential adverse event might facilitate the diagnosis for unexplained pericardial or pleural effusion after vaccination. Further studies are warranted to standardize the treatment and investigate the prognosis of this rare phenomenon.

## Data Availability

Not applicable.
